# Comparative Transcriptome Analysis Revealed Candidate Genes Potentially Related to Desiccation Sensitivity of Recalcitrant *Quercus variabilis* Seeds

**DOI:** 10.3389/fpls.2021.717563

**Published:** 2021-09-20

**Authors:** Dongxing Li, Yingchao Li, Jialian Qian, Xiaojuan Liu, Huihui Xu, Guowei Zhang, Junjie Ren, Libing Wang, Lu Zhang, Haiyan Yu

**Affiliations:** ^1^State Key Laboratory of Tree Genetics and Breeding, Research Institute of Forestry, Chinese Academy of Forestry, Beijing, China; ^2^Hongya Mountain State-Owned Forest Farm of Hebei, Yixian, China; ^3^College of Landscape and Architecture, Zhejiang Agriculture and Forestry University, Hangzhou, China; ^4^Zhejiang Provincial Key Laboratory of Germplasm Innovation and Utilization for Garden Plants, Zhejiang Agriculture and Forestry University, Hangzhou, China

**Keywords:** *Quercus variabilis*, recalcitrant seeds, desiccation sensitivity, transcriptome, differentially expressed genes, plant hormone signaling

## Abstract

Chinese cork oak (*Quercus variabilis*) is a widely distributed and highly valuable deciduous broadleaf tree from both ecological and economic perspectives. Seeds of this species are recalcitrant, i.e., sensitive to desiccation, which affects their storage and long-term preservation of germplasm. However, little is known about the underlying molecular mechanism of desiccation sensitivity of *Q. variabilis* seeds. In this study, the seeds were desiccated with silica gel for certain days as different treatments from 0 (Control) to 15 days (T15) with a gradient of 1 day. According to the seed germination percentage, four key stages (Control, T2, T4, and T11) were found. Then the transcriptomic profiles of these four stages were compared. A total of 4,405, 4,441, and 5,907 differentially expressed genes (DEGs) were identified in T2 vs. Control, T4 vs. Control, and T11 vs. Control, respectively. Among them, 2,219 DEGs were overlapped in the three comparison groups. Kyoto Encyclopedia of Genes and Genomes (KEGG) enrichment analysis showed that these DEGs were enriched into 124 pathways, such as “Plant hormone signal transduction” and “Glycerophospholipid metabolism”. DEGs related to hormone biosynthesis and signal transduction (*ZEP, YUC, PYR, ABI5, ERF1B*, etc.), stress response proteins (*LEA D-29, HSP70*, etc.), and phospholipase D (*PLD1*) were detected during desiccation. These genes and their interactions may determine the desiccation sensitivity of seeds. In addition, group specific DEGs were also identified in T2 vs. Control (*PP2C62, UNE12*, etc.), T4 vs. Control (*WRKY1-like, WAK10*, etc.), and T11 vs. Control (*IBH1, bZIP44*, etc.), respectively. Finally, a possible work model was proposed to show the molecular regulation mechanism of desiccation sensitivity in *Q. variabilis* seeds. This is the first report on the molecular regulation mechanism of desiccation sensitivity of *Q. variabilis* seeds using RNA-Seq. The findings could make a great contribution to seed storage and long-term conservation of recalcitrant seeds in the future.

## Introduction

Vegetative propagation is an extremely useful method for tree propagation. It can not only capture the superior phenotypes of selected trees but also propagates uniform clones (Pacholczak et al., 2017). However, many *Quercus* species have difficulties in vegetative propagation due to weak rooting ability, low survival, and growth ratio, etc. (Brennan et al., 2017; Li et al., 2019), and thus, their regeneration and reproduction mainly depend on seeds.

According to desiccation sensitivity, seeds are classified into orthodox, intermediate, and recalcitrant (Ellis et al., 1991). Unlike orthodox seeds, desiccation tolerance of recalcitrant seeds is not acquired during the late maturation stage, resulting in their sensitivity to desiccation. Therefore, recalcitrant seeds lose viability rapidly by using conventional storage methods (low moisture content and low temperature) (Walters et al., 2013; Obroucheva et al., 2016). Numerous plant species, especially for those naturally distributed in tropical and subtropical areas, produce recalcitrant seeds (Obroucheva et al., 2016). For example, seeds of many *Quercus* species have been demonstrated to be recalcitrant (Xia et al., 2015). Seed desiccation sensitivity is considered as an important functional and secondary evolutionary characteristic with a series of molecular processes being involved, such as gene expression regulation at the transcriptional level, phytohormone signaling transduction, and accumulation of late embryogenesis abundant proteins (LEAs) (Marques et al., 2018; Kijak and Ratajczak, 2020). In recalcitrant seeds, genes related to abscisic acid (ABA) biosynthesis and signal transduction, as well as LEAs and heat shock proteins (HSPs) were found to be differentially expressed during desiccation (Wei et al., 2017; Jin et al., 2018; Marques et al., 2018). The acquisition of seed desiccation tolerance is closely related to multiple genes related to ABA, LEAs, and HSPs (González-Morales et al., 2016). For example, ABSCISIC ACID INSENSI-TIVE3 (ABI3) is one of four major regulators of seed desiccation tolerance acquisition in *Arabidopsis thaliana*, and its downstream transcription factors play crucial roles in the desiccation tolerance acquisition network (Roscoe et al., 2015). Furthermore, desiccation tolerance acquisition of *Coffea arabica* seed was concomitant with a decrease of indole-3-acetic-acid (IAA) content (Dussert et al., 2018). Several genes that control the homeostatic regulation and inactivation of IAA level, i.e., genes encoding for indole-3-acetic acid-amido synthetase GH3.3 and uridine diphosphate (UDP)-glycosyltransferase 74E2 (Dussert et al., 2018) were induced during desiccation tolerance acquisition. It has been reported that the UDP-glucosyltransferase UGT74E2 modulate water stress tolerance in *Arabidopsis* (Tognetti et al., 2010).

Transcriptome analysis can reflect the gene expression level and has been used for investigating molecular mechanisms of the response of a plant under abiotic stress (Velculescu et al., 1997; Zhang et al., 2017a,b). For instance, the popular RNA-Seq technology has been used to explore the underlying mechanisms of desiccation sensitivity of recalcitrant seeds in some species such as *Citrus limon* and *Taxillusi chinensis* (Wei et al., 2017; Marques et al., 2019). However, these species produce relatively small seeds, which lead them to be atypical recalcitrant seeds. Furthermore, these studies did not integrate the transcriptome analysis with important physiological characteristics, especially the hormone content. Therefore, the molecular mechanisms of desiccation sensitivity are largely unclear.

*Quercus variabilis* Blume is an important afforestation and economic timber species, with high ecological and economic value (Yuan et al., 2020). Its bark is widely used for making wine bottle stoppers, heat preservation, and environmental protection (Gil, 2014). Its acorns are rich in nutrition and high in starch, which can be used not only as raw materials for food, medicine, and wine making (Guamán-Guamán and Willlams-Linera, 2006), but also to produce the fuel, ethanol. However, *Q. variabilis* seeds are typical recalcitrant seeds. High sensitivity to water loss is the main factor affecting its seed storage and long-term preservation of germplasm resources. Hence, the purpose of this research was to explore the physiological responses (ABA and IAA) and gene expression changes during the desiccation of *Q. variabilis* seeds by RNA-Seq, then identify desiccation-responsive genes and analyze the potential molecular mechanism of desiccation sensitivity regulation. The results will not only provide insights into the molecular regulation mechanism of seeds but will also contribute to seedling breeding, seed storage, and long-term conservation of germplasm resources in *Q. variabilis*.

## Materials and Methods

### Plant Materials

Mature seeds were collected from one *Q. variabilis* tree in September 16, 2019 located in Louguantai Forest Farm (Shanxi, China) and no specific permissions were required to collect these seeds. Then, they were confirmed by Prof. Fang Du and were tested in the laboratory of Chinese Academy of Forestry. The impurities and inferior seeds were first removed by visual check. Then, the seeds were immersed in water for 5 min to separate viable seeds from floating seeds (Liu et al., 2012). The viable seeds were air-dried in the shade for 12–15 h. During that period, the seeds were turned every 2–3 h. Finally, the healthy, uniform, and non-germinating seeds were used for desiccation experiments.

### Determination of Seed Moisture Content

A random of 20 seeds × 3 replications were selected for computation of initial moisture content. The initial moisture content of *Q. variabilis* seeds (Control) was determined as described in Ganatsas and Tsakaldimi (2013). The moisture content of seeds was calculated by initial moisture content minus that of desiccation.

### Desiccation Treatments

Forty-five seeds with three replications were selected for per desiccation treatment. These seeds and silica gel (the mass ratio of seeds to silica gel was about 1:3) were placed in zip-lock bags for 0 day (Control), 1 day (T1) to 15 days (T15). Then, the desiccation test was conducted in laboratory conditions with a temperature of 20 to ~25°C and relative humidity of 40 to ~45%. Seed samples were collected on 10 seeds with three replications per desiccation treatment. The samples were frozen in liquid nitrogen and stored at −80°C for further analyses.

### Seed Germination

After desiccation, seed germination was tested. There were four replicates in each treatment, with 25 seeds in each replicate. Seeds were placed on top of two pieces of filter paper and a layer of gauze moistened with deionized water in 12 cm diameter Petri dishes. Afterwards, Petri dishes were transferred to cyclically alternating temperature (30/20°C for 8/16 h, light/dark photoperiod) in a light incubator (Ganatsas and Tsakaldimi, 2013). The seeds were considered to have germinated when the emerging radicle was at least 2 mm (Ganatsas et al., 2017). Germination was recorded every day and tallied for up to 28 days (Kristinaf and Sharon, 2003). The germination percentage was calculated according to the following formula:


$$\begin{array}{l} GP=\Sigma Dt/N\times \;100\% \end{array}$$

where *Dt* denotes the number of germinations on *t* day, and *N* is the total number of seeds.

Finally, according to the germination assay, four critical periods (Control, T2, T4, and T11) of *Q. variabilis* seeds desiccation sensitivity were used for hormone measuring and transcriptome sequencing.

### Determination of ABA and IAA Concentration

Seed samples (50 mg fresh weight) were dissolved in 1 ml mixed solution with methanol/water/formic acid (15:4:1, V/V/V). The combined extracts were evaporated until dry under a stream of nitrogen, reconstituted in 80% methanol (V/V) and filtrated (PTFE, 0.22 μm; Anpel). Phytohormones contents were detected and analyzed by Metware Biotechnology Co., Ltd. (Wuhan, China, http://www.metware.cn/) based on the AB Sciex QTRAP 6500 LC-MS/MS platform. Three replicates were performed for measurement.

### Extraction, Transcriptome Sequencing, and Analysis of Total RNA From Seeds

Total RNA was extracted from seeds using a total RNA purification kit (TRK1001, LC Science, Houston, TX) following the manufacturer's procedure. The total RNA quantity and purity were analyzed by Agilent 2100 Bioanalyzer and RNA 6000 Nano LabChip Kit (Agilent, CA, USA), with RIN number > 8. Approximately, 10 μg of total RNA representing a specific adipose type was subjected to isolate Poly (A) mRNA with poly-T oligo attached magnetic beads (Invitrogen). Following purification, the mRNA was fragmented into small pieces using divalent cations under elevated temperature. Then the cleaved RNA fragments were reverse-transcribed to create the final cDNA library in accordance with the protocol for the mRNA-Seq sample preparation kit (Illumina, San Diego, USA). The average insert size for the paired-end libraries was 300 bp (± 50 bp). And then the paired-end sequencing was performed on an Illumina Hiseq 4000 at the (LC Sciences, USA) following the recommended protocol of the vendor. The CutAdapt (version 1.11, Martin, 2011) was used to remove low-quality reads and adaptor sequences. The clean data was aligned to the *Q. suber* genome (https://www.ncbi.nlm.nih.gov/genome/66905, Ramos et al., 2018) using HISAT (version 2.0, Kim et al., 2015). The mapped reads of each sample were assembled using String Tie (version 1.3.0, Pertea et al., 2015) and the Bioconductor edgeR (Robinson et al., 2010) was used to identify DEGs. Ultimately, GO and KEGG enrichment analyses were performed using the OmicStudio tools at https://www.omicstudio.cn/tool. The *p* < 0.05 were defined as significantly enriched items and pathways.

### Quantitative Real-Time PCR Analysis

Nine DEGs with potential functions in regulating desiccation sensitivity of *Q. variabilis* seeds were selected for qRT-PCR validation. Total RNA was reversely transcribed into cDNA by using Prime Script™ Reagent Kit with gDNA Eraser (Takara, Dalian, China). The qRT-PCR assay was performed with KAPA SYBR FAST qPCR Master Mix (Kapa Bio-systems, USA), according to the instructions of the manufacturer. The primers used for qRT-PCR were designed using Premier-BLAST (Goh et al., 2019) and premier sequences were listed in Supplementary Table S1. The thermal cycling conditions were as follows: 95°C for 30 s, 40 cycles of 5 s at 95°C, and 60°C for 30 s. *ACTIN* was used as the reference gene (Marum et al., 2012) and the relative expression data was calculated by using the 2^−ΔΔCt^ method (Livak and Schmittgen, 2001).

### Statistical Analysis

The significance among the means of physiological characteristics was verified using ANOVA, followed by Duncan's test at *p* ≤ 0.05. Pearson's correlation test was conducted to calculate the relationships between germination percentage, moisture content, ABA, and IAA contents. All statistical analysis were finished by SPSS 22.0.

## Results

### Seed Germination Percentage

**Figure 1:**
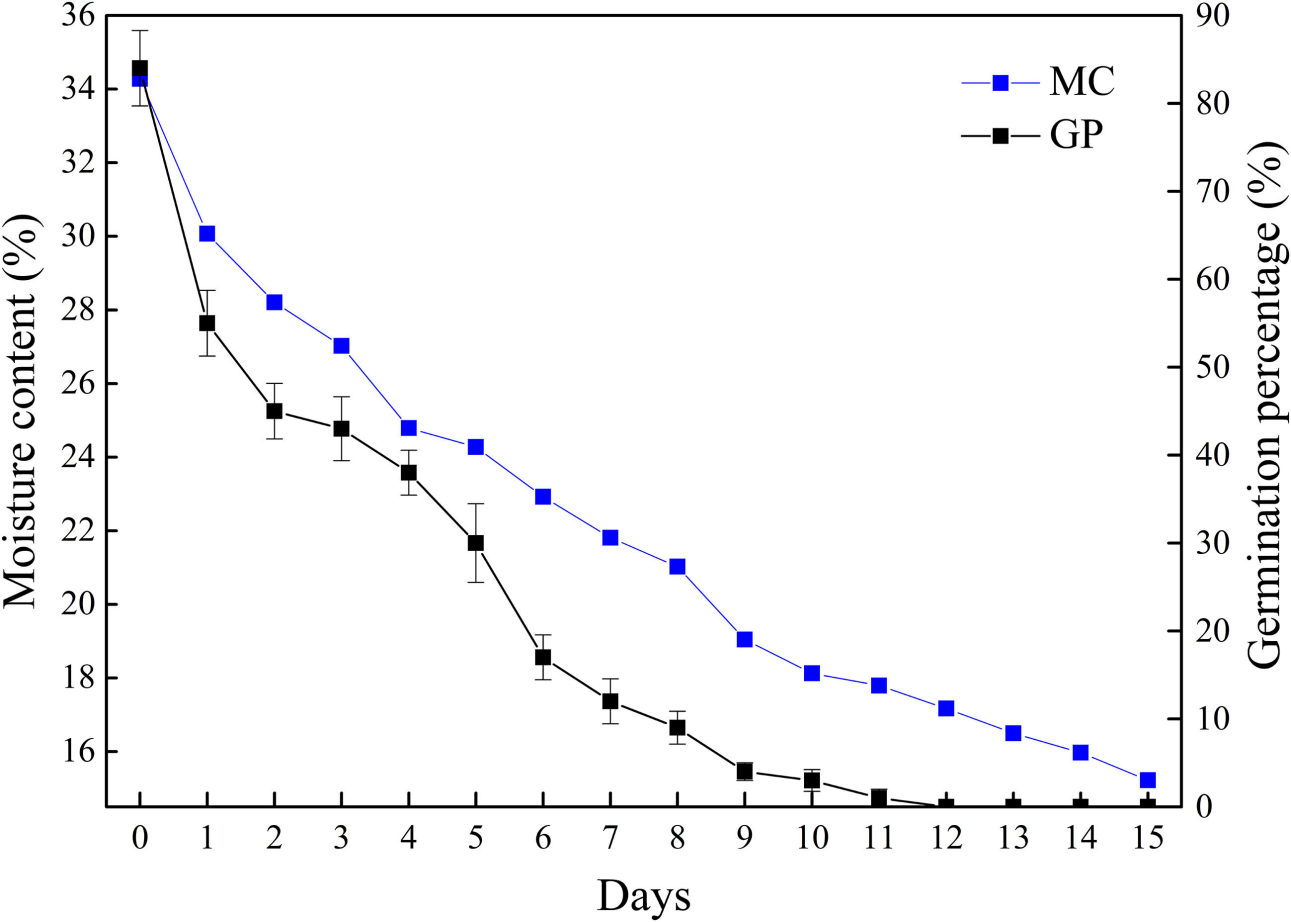
Changes of germination percentage and moisture content of *Q. variabilis* seeds during desiccation. The numbers in the horizontal axis represent days after desiccation treatment. Vertical lines represent ± SE of the means.

The moisture content and germination percentage of *Q. variabilis* seeds decreased with the desiccation time (Figure 1). The germination percentage and moisture content of seeds in the control group were 84 and 34.27%, respectively. At the early stage of desiccation (T1–T2), the germination percentage decreased rapidly. After a slow decline stage (T2–T4), the germination percentage decreased rapidly again (T4–T6), then gradually decreased to 0% (T12). Meanwhile, the moisture content reduced to 17.17% after 12 days. In addition, the moisture content of seeds was significantly positively correlated with germination percentage (*p* < 0.01, *R*^2^ = 0.976). These results indicate that *Q. variabilis* seeds are highly sensitive to desiccation.

### ABA and IAA Contents

**Figure 2:**
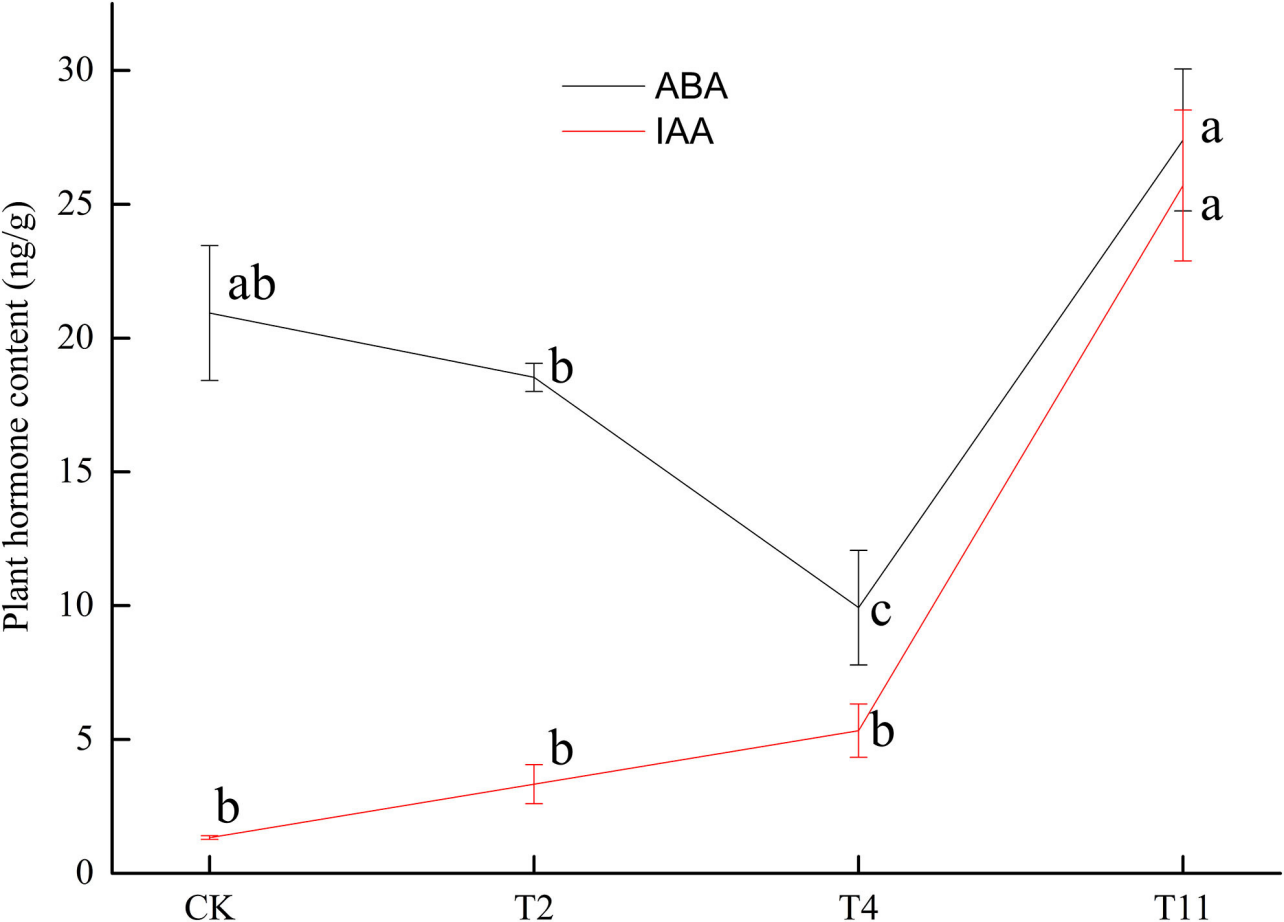
Changes of plant hormone [abscisic acid (ABA) and indole-3-acetic acid (IAA)] content in *Q. variabilis* seeds during desiccation. Vertical lines represent ± SE of the means. Different lowercase letters represent significant differences between different treatments at the 0.05 probability level. Control (CK), desiccation for 0 day; T2, desiccation for 2 days; T4, desiccation for 4 days; T11, desiccation for 11 days.

The ABA content decreased from 20.93 ± 2.51 ng/g (Control) to 18.53 ± 0.52 ng/g (T2), then to 9.93 ± 2.13 ng/g (T4) and finally increased to 27.40 ± 2.65 ng/g (T11). Meanwhile, IAA content showed a rising trend from 1.34 ± 0.07 ng/g (Control) to 25.70 ± 2.82 ng/g (T11) (Figure 2). In addition, there was a significantly negative correlation (*p* < 0.01, *R*^2^= −0.87) between moisture content and IAA content during seed desiccation (Supplementary Figure S1). And there was also a significantly negative correlation (*p* < 0.01, *R*^2^ = −0.81) between germination percentage and IAA content during this process (Supplementary Figure S2). However, it was found that there was insignificant correlation (*p* > 0.05) between moisture content, germination percentage, and ABA content (Supplementary Figures S3, S4).

### Analysis of Illumina Sequencing Results

A total of 629.44 million raw reads were obtained from 12 libraries. Ultimately, 596.16 million clean reads were obtained after filtering out low-quality reads, adaptor and N-containing sequences, with GC contents and Q30 values ranged from 43.50 to 44.00 and 97.85 to 98.28%, respectively (Supplementary Table S2). To align the clean reads to the reference genome (GCF_002906115.1), 86.73–88.33% of clean reads were mapped and 50.83–52.61% were uniquely mapped (Supplementary Table S2).

### Identification DEGs During Desiccation

**Figure 3:**
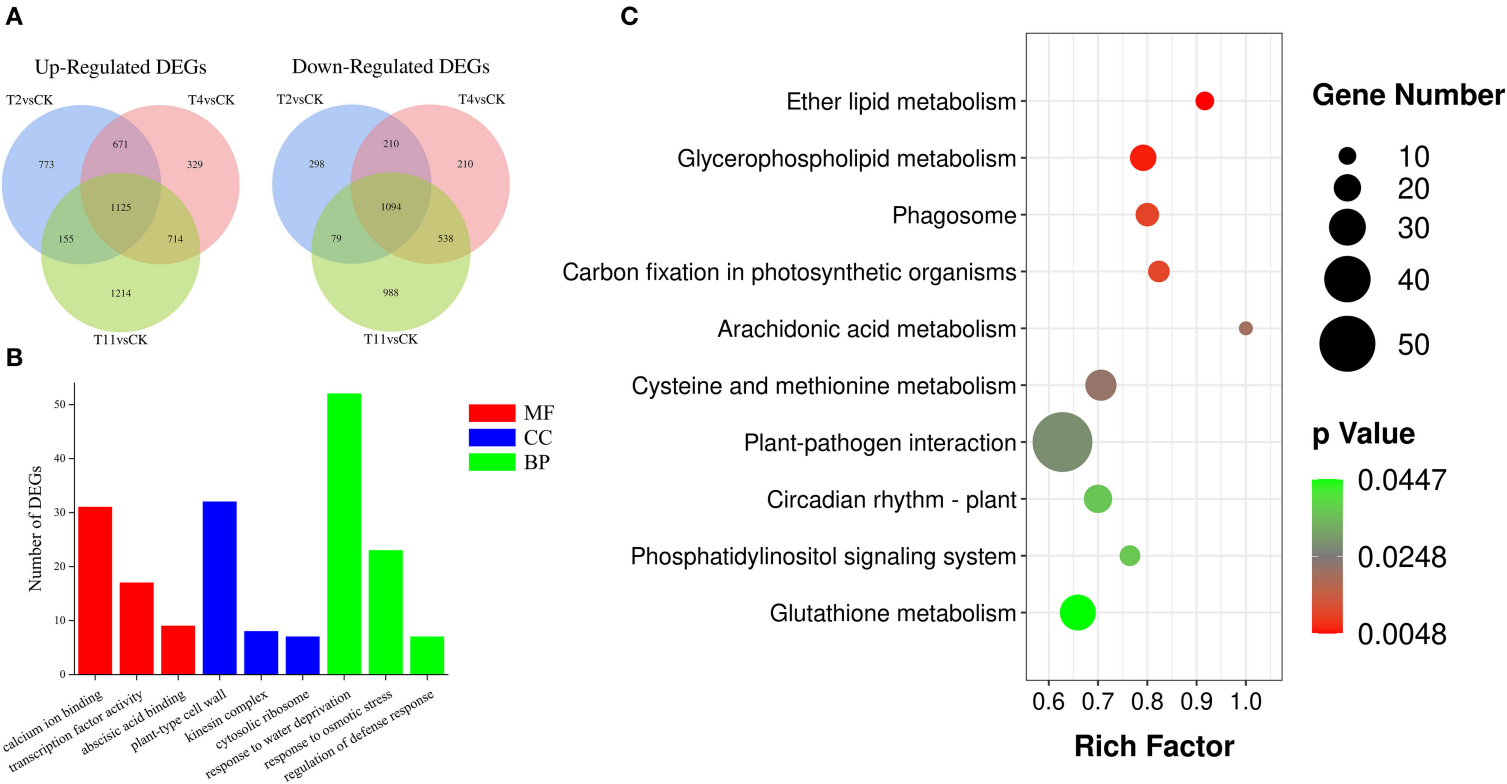
The number of differentially expressed genes (DEGs) identified by comparisons and enrichment analysis. **(A)** Venn diagrams show the number of up and downregulated DEGs in three comparison groups, **(B)** Gene Ontology (GO) enrichment analysis of DEGs, including molecular function, cellular component and biological process, **(C)** Kyoto Encyclopedia of Genes and Genomes (KEGG) enrichment analysis of DEGs.

To better investigate gene expression changes during seed desiccation, three comparison groups were established: T2 vs. Control, T4 vs. Control, and T11 vs. Control. In total, 4,405 (2,724 up regulated and 1,681 downregulated), 4,441 (2,839 up regulated and 2,052 downregulated), and 5,907 (3,208 up regulated and 2,699 downregulated) DEGs were identified in these groups by using fragments per kilobase of transcript per million (FPKM) (|log_2_ (fold change)| ≥ 1 and *Padj* < 0.05), respectively (Figure 3A). In the three comparison groups, the same DEGs (1,125 up regulated and 1,094 downregulated) were found (Supplementary Table S3). Additionally, 1,071 (773 up regulated and 298 downregulated), 539 (329 up regulated and 210 downregulated) and 2,202 (1,214 up regulated and 988 downregulated) specific DEGs were found in T2 vs. Control, T4 vs. Control, and T11 vs. Control, respectively (Figure 3A; Supplementary Table S4).

### Gene Ontology (GO) and KEGG Analyses of DEGs During Desiccation

To explore biological functions of the common DEGs in all three comparison groups in *Q. variabilis* seeds during desiccation, GO enrichment analysis was conducted. The results showed that these DEGs covered three GO categories: “Molecular function (MF)”, “Cellular component (CC)”, and “Biological progress (BP)”. Within the MF categories, the DEGs were significantly involved in “Calcium ion binding”, “Transcription factor activity”, and “Abscisic acid binding”. As for the GO terms of CC, the DEGs were significantly included in “Plant-type cell wall”, “Kinesin complex”, and “Cytosolic ribosome”. In the BP categories, the DEGs were significantly enriched in “Response to water deprivation”, “Response to osmotic stress”, and “Regulation of defense response” (Figure 3B; Supplementary Table S5). In addition, the group specific DEGs were also subjected to GO enrichment analysis. In T2 vs. Control, the specific DEGs were involved in “Signal transduction”, “Response to abscisic acid”, “Response to auxin”, and “Protein serine/threonine kinase activity”. In T4 vs. Control, specific DEGs were enriched in “Response to stress”, “Transcription factor activity”, and “Protein phosphorylation”. Lastly, for T11 vs. Control group, specific DEGs were included in “Response to water deprivation”, “Response to abscisic acid”, and “Kinase activity” (Supplementary Figure S5).

To further characterize the DEG-associated metabolism pathways, the common DEGs in all three comparison groups were subjected to KEGG enrichment analysis. It is found that these DEGs were involved in 124 pathways. And most of them were enriched in the pathway of “Protein processing in endoplasmic reticulum” (ko04141), followed by “Plant hormone signal transduction” (ko04075) and “Plant pathogen interaction” (ko04626) (Supplementary Table S6). The significantly enriched pathways were mainly involved in “Ether lipid metabolism” (ko00565), “Glycerophospholipid metabolism” (ko00564), “Carbon fixation in photosynthetic organisms” (ko00710), and others (Figure 3C). In addition, the group specific DEGs were also subjected to KEGG enrichment analysis. In T2 vs. Control, the specific DEGs were enriched in “Phenylpropanoid biosynthesis” (ko00940), “Endocytosis” (ko04144) and others. In T4 vs. Control, specific DEGs were involved in “Phenylpropanoid biosynthesis” (ko00940), “Pentose and glucuronate interconversions” (ko00040), and other pathways. In T11 vs. Control, specific DEGs were included in “RNA transport” (ko03013), “Glutathione metabolism” (ko00480), and others (Supplementary Figure S6). The analysis illustrated that there are different response strategies of *Q. variabilis* seeds at early, middle, and late desiccation stages.

### DEGs Related to Plant Hormones

**Figure 4:**
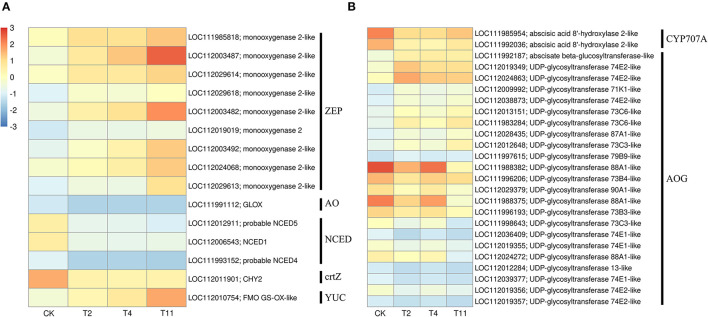
Heat maps show the effects of desiccation on plant hormone biosynthesis and catabolism in *Q. variabilis* seeds. **(A)** The expression of DEGs that might be involved in “ABA and IAA biosynthesis” on different days after desiccation treatment. **(B)** The expression of DEGs that might be involved in “ABA catabolism” on different days after desiccation treatment. Control (CK), desiccation for 0 day; T2, desiccation for 2 days; T4, desiccation for 4 days; T11, desiccation for 11 days. The log10(FPKM+1) is used to normalize FPKM in heatmap.

**Figure 5:**
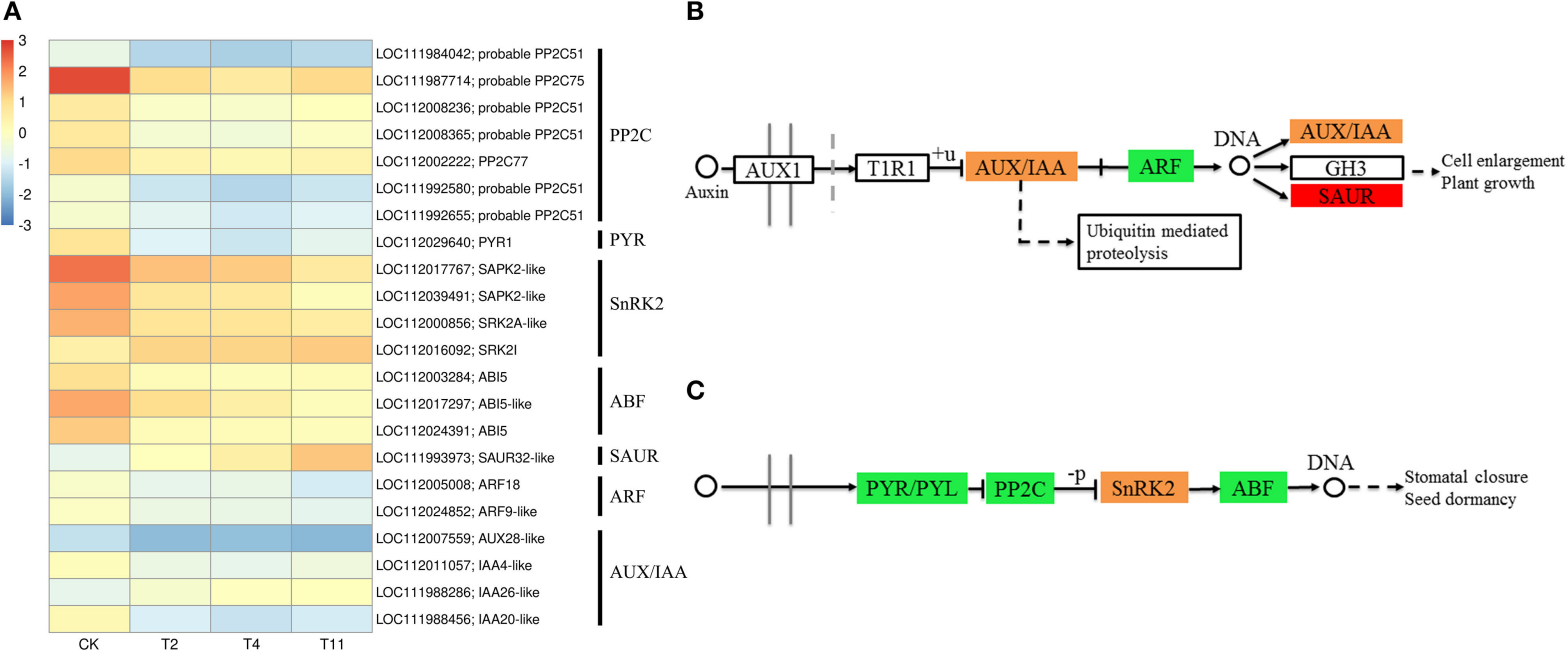
Effects of desiccation on the expression of genes in ABA and IAA signal transduction pathway. Heat maps shows DEGs related to ABA and IAA signal transduction pathway **(A)**, **(B)**, and **(C)** show the pathways of IAA and ABA signaling. The boxes represent regulatory genes, and circles represent metabolites. Red indicates that the gene expression is up regulated, green indicates that the gene expression is downregulated, while orange indicates that there are both up regulated and downregulated genes in the gene family. Control (CK), desiccation for 0 day; T2, desiccation for 2 days; T4, desiccation for 4 days; T11, desiccation for 11 days. The log10(FPKM+1) is used to normalize FPKM in heatmap.

**Figure 6:**
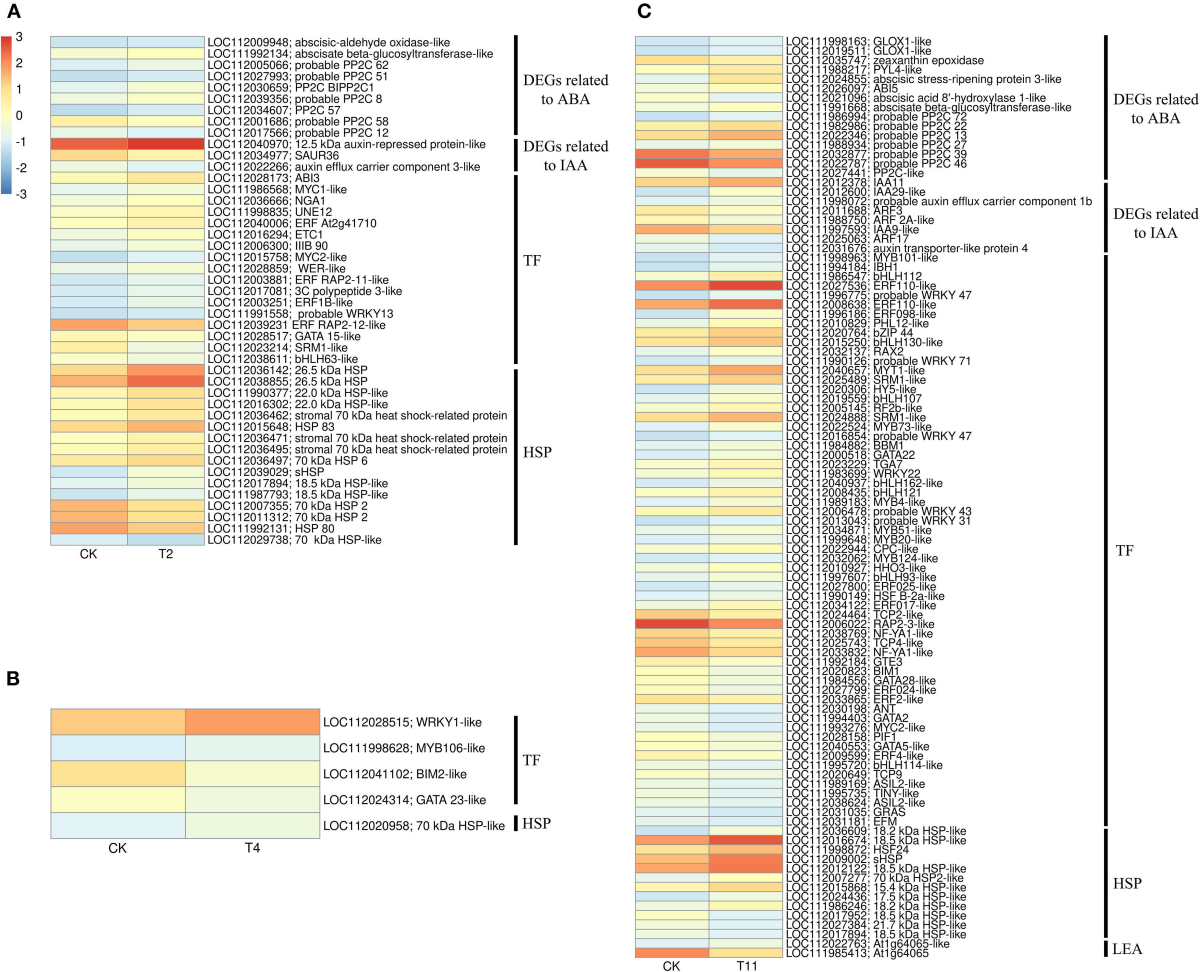
Heat maps show that the group specific DEGs in three comparison groups. **(A–C)** indicate the comparison group of T2 vs. Control, T4 vs. Control, and T11 vs. Control, respectively. Control (CK), desiccation for 0 day; T2, desiccation for 2 days; T4, desiccation for 4 days; T11, desiccation for 11 days. The log10(FPKM+1) is used to normalize FPKM in heatmap.

In the common DEGs, in all three comparison groups, genes encoding beta-carotene 3-hydroxylase (crtZ), 9-cis-epoxycarotenoid dioxygenase (NCED), and aldehyde oxidase (AO) in ABA biosynthesis were downregulated during seed desiccation. Two genes encoding abscisic acid 8′-hydroxylases (CYP707A) and 13 encoding ABA β- and UDP glucosyltransferase (AOG) related to ABA catabolism were downregulated during desiccation. Meanwhile, genes encoding zeaxanthin epoxidase (ZEP) and ten AOG genes were up regulated during desiccation (Figures 4A,B). Genes related to ABA signaling transduction were differentially expressed during seed desiccation. The ABA receptor PYR/PYL family (*PYR*), Protein phosphatase 2C (*PP2C*), three Sucrose Non-fermenting 1-related protein kinase 2 (*SnRK2*) and ABA responsive element binding factor (*ABF*) genes were downregulated, while one *SnRK2* gene was up regulated (Figures 5A,C). Furthermore, group specific DEGs were also identified as related to ABA in T2 vs. Control (*AO, AOG, PP2C*) and T11 vs. Control (*AO, ZEP, CYP707A*, etc.), respectively (Figures 6A,C). But group specific DEGs related to ABA in T4 vs. Control was not found.

At the same time, one gene (LOC112010754) encoding flavin containing monooxygenase (FMO) related to IAA biosynthesis was up regulated continuously during desiccation (Figure 4A). Furthermore, desiccation also interfered the IAA signal transduction pathway. During desiccation, one gene encoding auxin response protein (AUX/IAA) and one SAUR gene were up regulated. Meanwhile, three AUX/IAA and two genes encoding auxin response factor (ARF) were downregulated (Figures 5A,B). In addition, group specific DEGs were also found to be related to IAA in T2 vs. Control (AUX/IAA, SAUR) and T11 vs. Control (AUX/IAA, SAUR, ARF), respectively (Figures 6A,C). But group specific DEGs related to IAA in T4 vs. Control was not found.

### DEGs Related to Transcription Factors and Dehydrating Proteins

**Figure 7:**
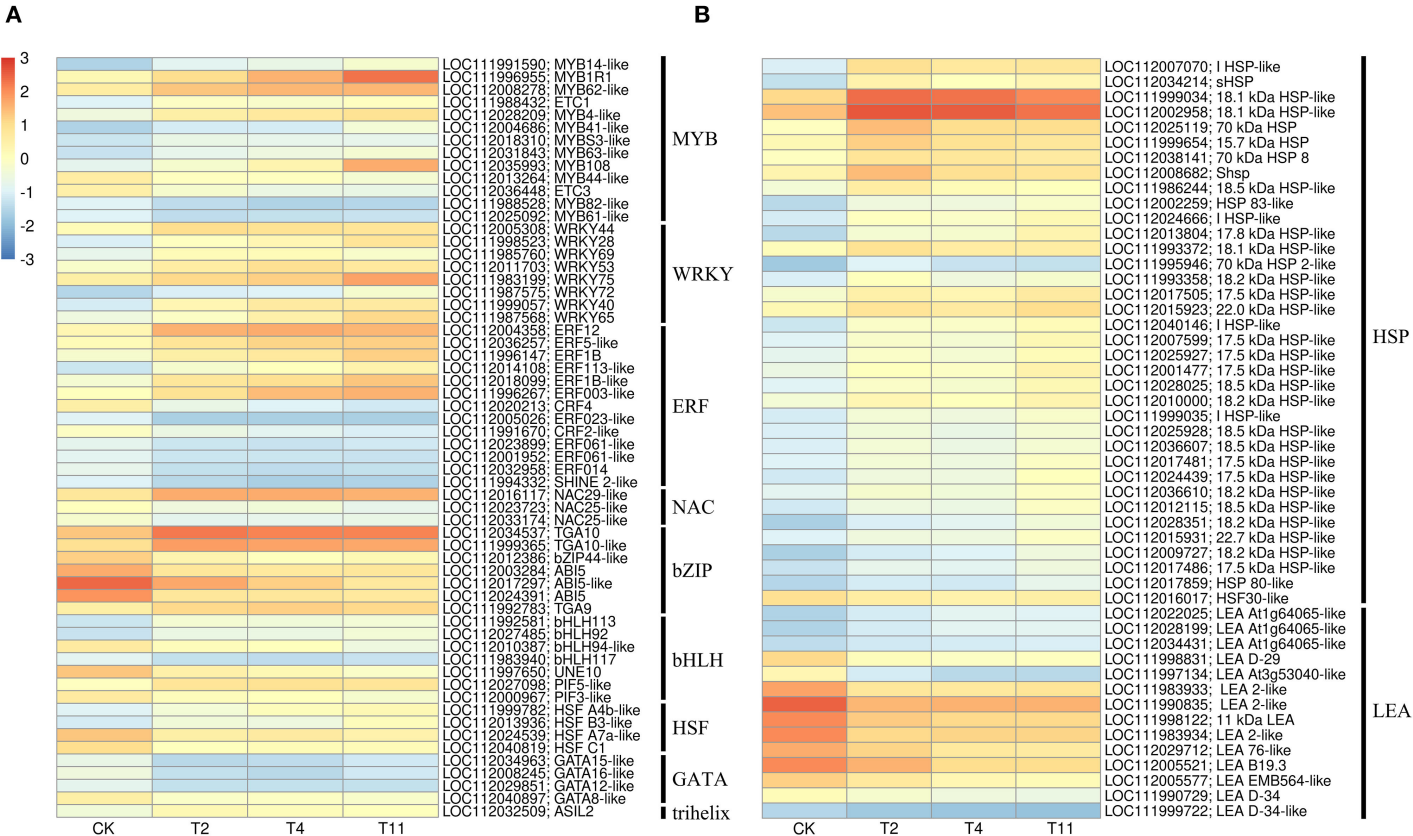
Effects of desiccation on gene expression of transcription factors and protective proteins. Heat maps show that the expression of DEGs related to transcription factor **(A)** and protective protein **(B)**, respectively on different days after desiccation treatment. Control (CK), desiccation for 0 day; T2, desiccation for 2 days; T4, desiccation for 4 days; T11, desiccation for 11 days. The log10(FPKM+1) is used to normalize FPKM in heatmap.

**Figure 8:**
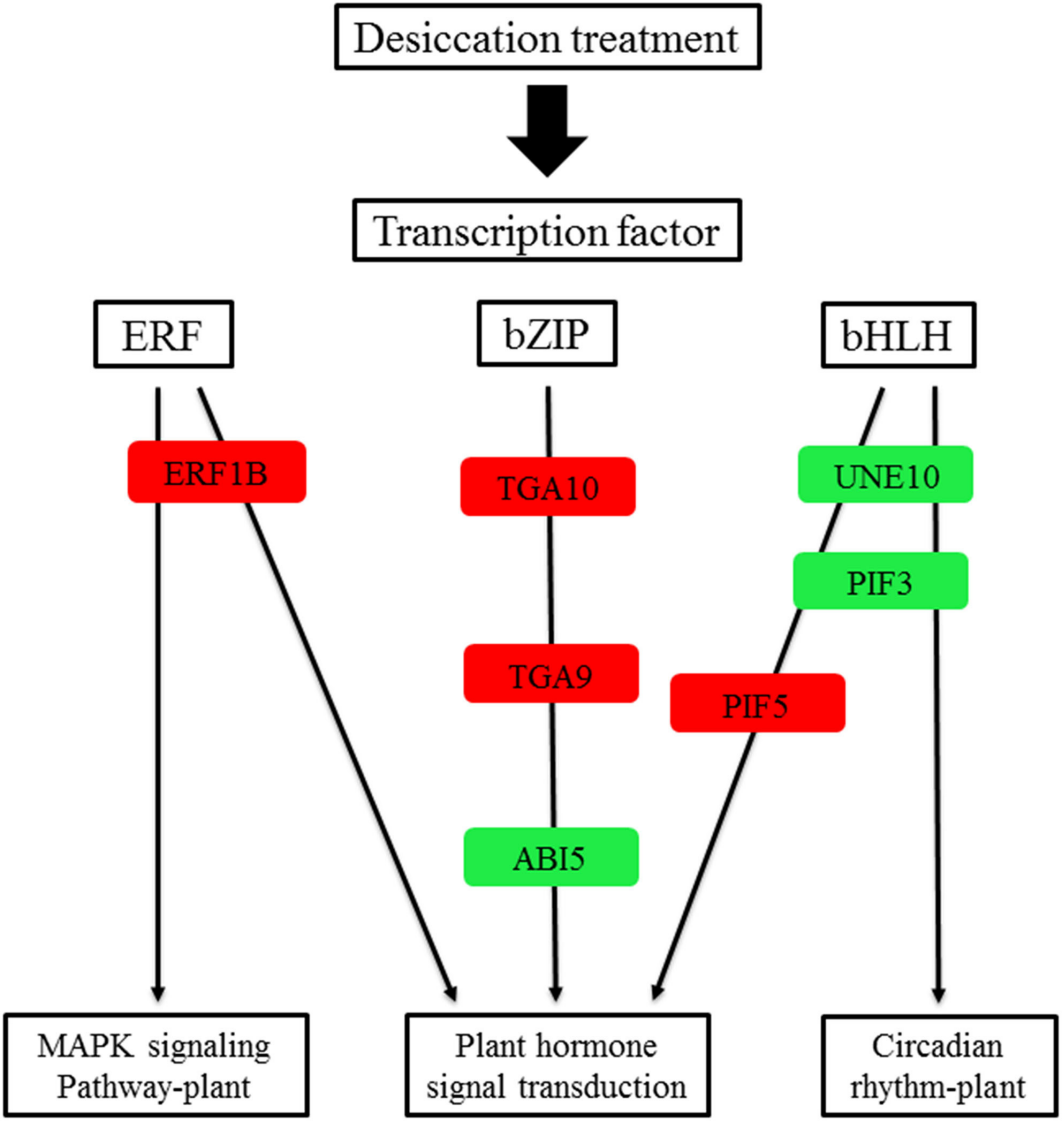
A proposed working model for desiccation transcription factor signaling network in *Q. variabilis* seeds. Red indicates that the gene expression is up regulated, green indicates that the gene expression is downregulated.

In the common DEGs, in all three comparison groups, differentially expressed transcription factors (TFs) in *Q. variabilis* seeds, under desiccation, were mainly distributed in nine families: MYB, WRKY, ERF, NAC, bZIP, bHLH, Hsfs, GATA, and Trihelix (Figure 7A). For example, the TFs of bZIP family (*TGA10, TGA9*) were up regulated and *ABI5* were downregulated during desiccation. The ERF1B may represent the crucial protein mediating crosstalk between “MAPK signaling pathway-plant” and “Plant hormone signal transduction” in response to desiccation. The UNE10 and PIF3 may be important proteins mediating crosstalk between “Plant hormone signal transduction” and “Circadian rhythm-plant” during desiccation (Figure 8). In addition, the group specific TFs were also found. There were 17 (13 up regulated and 4 downregulated), 4 (2 up regulated and 2 downregulated), and 61 (38 up regulated and 23 downregulated) TFs in three comparison groups, respectively (Figures 6A–C).

In this study, 36 and 14 genes encoding HSPs and LEAs were differentially expressed during desiccation, respectively (Figure 7B). Except for HSF30-like (*LOC112016017*), HSPs related genes were all up regulated, while LEAs-related genes, except for At1g64065-like (*LOC11202022025, LOC112028199* and *LOC112034431*), were all downregulated (Figure 7B). Additionally, 16, 1, and 12 group specific DEGs related to HSPs were identified in T2 vs. Control, T11 vs. Control, and T11 vs. Control, respectively. And two group specific DEGs related to LEAs were found only in T11 vs. Control (Figure 6C).

### DEGs Related to Glycerophospholipid Metabolism

**Figure 9:**
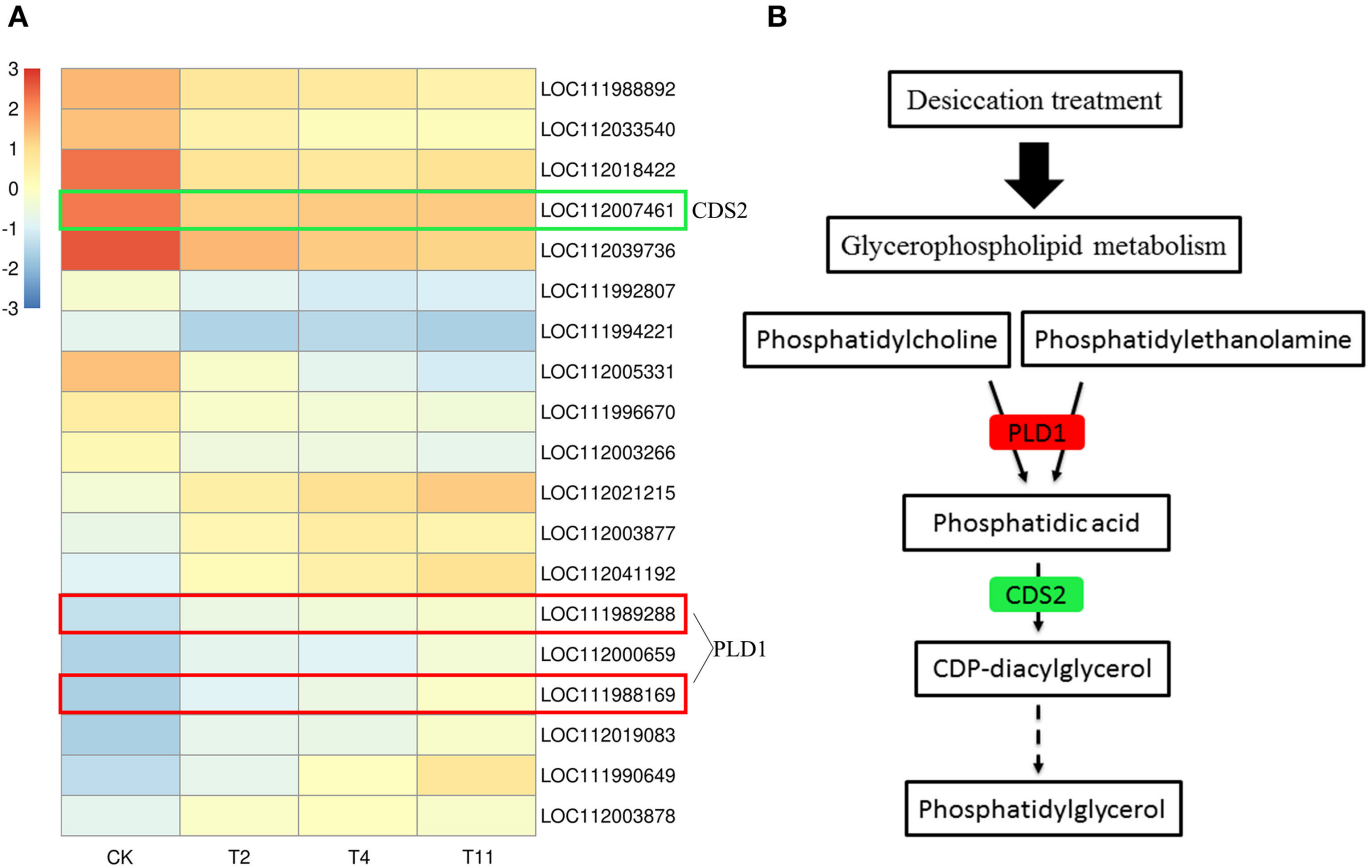
Gene expression level in glycerophospholipid metabolism pathway after treatment by desiccation. Heat map illustrates DEGs related to glycerophospholipid metabolism pathway **(A)**. Partial pathway of glycerophospholipid metabolism **(B)**. Red indicates that the gene expression is up regulated, green indicates that the gene expression is downregulated. Control (CK), desiccation for 0 day; T2, desiccation for 2 days; T4, desiccation for 4 days; T11, desiccation for 11 days. The log10(FPKM+1) is used to normalize FPKM in heatmap.

Glycerophospholipid is the most abundant phospholipid in the organism and is an important component of membrane. In the common DEGs in all three comparison groups, nine up regulated and ten downregulated DEGs in glycerophospholipid metabolism pathway were found (Figure 9A). Among them, the expression of *PLD1* was upregulated and that of *CDS2* was downregulated (Figure 9B).

### Validation of DEGs by qRT-PCR

**Figure 10:**
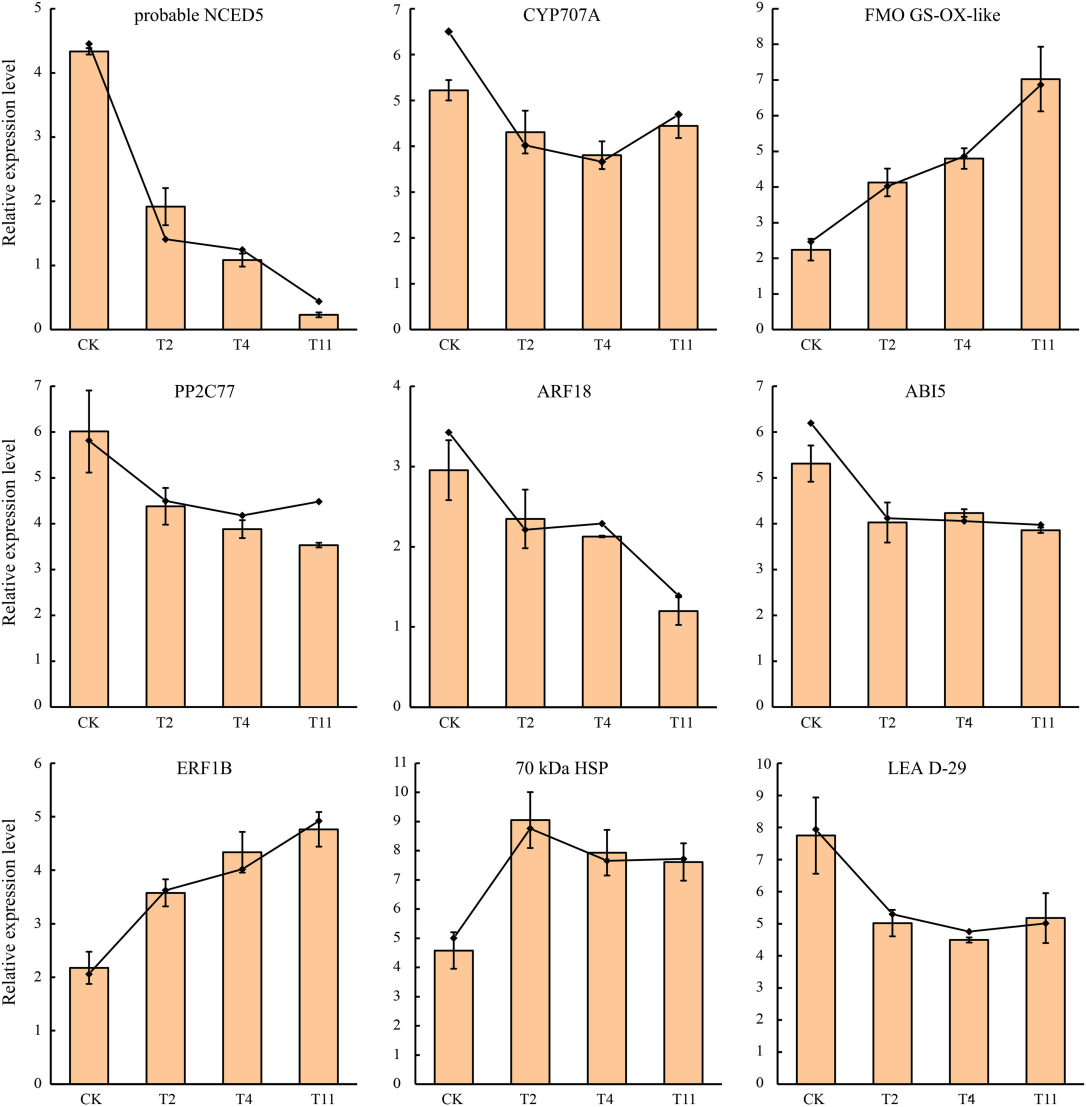
Expression patterns of nine selected DEGs in *Q. variabilis* seeds according to qRT-PCR and RNA-Seq. The broken line indicates RNA-Seq data, while histogram indicates qRT-PCR data. Vertical lines represent ± SE of the means.

To confirm the RNA-Seq data, the expression quantification of nine DEGs were validated by qRT-PCR. Although the |log_2_ fold change| values of these DEGs were different from RNA-Seq, the patterns were similar (Figure 10), suggesting that the RNA-Seq results were reliable.

## Discussion

### Effect of Desiccation on Seed Germination

Generally, orthodox seeds can tolerate drying and have a high germination percentage (Kijak and Ratajczak, 2020). For example, when the moisture content decreased to 8%, the germination percentage of *Arabidopsis* seeds was maintained at nearly 100% (Jing et al., 2018). For the *Pisum sativum* seeds, the total level of germination percentage was maintained above 90% during desiccation (Chen et al., 2019). However, the germination percentage of *Q. variabilis* seeds decreased along with the decrease of moisture content in this study, which is consistent with other recalcitrant seeds (Ganatsas and Tsakaldimi, 2013; Feng et al., 2017; Wei et al., 2017; Jin et al., 2018). There was a significantly positive correlation between moisture content and germination percentage. The critical moisture content of recalcitrant seeds is often determined when about 50% of the seeds become unviable, which can be used as a standard of seed desiccation sensitivity (Obroucheva et al., 2016). It has been reported that the critical moisture content of *Ginkgo biloba* seeds is between 45 and 40.10% (Feng et al., 2017). However, in the present study, the critical moisture content of the seed was about 28.20%. Different critical moisture content may be due to the interspecific differences. Furthermore, when the moisture content of seeds decreased to 17.17%, the seeds lost their vitality completely, which was similar to the recalcitrant seeds of *Aesculus chinensi* (Yu et al., 2006).

### Plant Hormone Biosynthesis

In plants, hormones play important roles in coping with abiotic stress (Chen et al., 2019). For in-stance, ABA can regulate the drought stress response and is also a vital hormone for seed desiccation tolerance acquisition (Khandelwal et al., 2010). The ABA biosynthesis and catabolism in plants are mainly regulated by *NCED* and *CYP707A* (Son et al., 2016). Similar to a previous study on recalcitrant *Camellia sinensis* seeds (Jin et al., 2018), genes related to ABA biosynthesis, such as *NCED, crtZ*, and *AO* were downregulated, while *ZEP* was up regulated during desiccation. Furthermore, previous research has also pointed out that the lack of ABA in the ZEP-deficient mutant of *Nicotiana plumbaginifolia* resulted in rapid water loss (Marin et al., 1996). In this study, although the expression of *ZEP* was up regulated, the downregulation of *NCED, crtZ* and *AO* might lead to the decrease of ABA content during seed early (T2) and middle (T4) desiccation stages. Interestingly, *NCED, AO* and *crtZ* had increased expressions at the late desiccation stage (T11). They may work together with the up regulated *ZEP* to increase ABA content. Furthermore, ABA 8′-hydroxylases encoded by *CYP707A* can deactivate ABA and play an irreplaceable role in ABA catabolism (Kushiro et al., 2004). The ABA can also be reversibly inactivated by glycosylation. For instance, plants lacking UDP glucosyltransferase have fewer glucosyl esters and freer ABA, exhibiting stronger resistance to water deficit (Liu et al., 2015). In the study, genes encoding ABA β-glucosyltransferase were downregulated, but their expression were increased at late seed desiccation stage (T11). And genes encoding UDP glycosyltransferase were both up and downregulated. Therefore, it was speculated that the downregulation of genes encoding NCED, crtZ, AO, ABA β-glucosyltransferase, and the upregulation of UDP glycosyltransferase related genes might cause the decline of ABA content during early and middle seed desiccation stages. The up regulated ZEP, downregulated UDP glycosyltransferase related genes, and change in gene expression at the late seed desiccation stage may cause the upward change of ABA level, which is similar to the recalcitrant seeds of *T. chinensis* (Wei, 2017). In addition, it is widely known that indole pyruvate pathway is the most basic pathway for IAA biosynthesis in plants (Zhao, 2012). The FMO encoded by YUC family genes has been confirmed to be able to directly convert indole pyruvate to IAA (Mashiguchi et al., 2011). In this study, it was found that YUC family gene (*LOC112010754*) was up regulated, which may lead to the increase of IAA content during desiccation.

Abscisic acid and IAA are closely related to many physiological and developmental processes such as seed dormancy (Liu et al., 2013), lateral root development (Zhao et al., 2014), and plant growth (Wang et al., 2011). It has been reported that the upregulated expression of many genes related to IAA may inhibit the mitochondrial retrograde response while ABA has the opposite effect, playing an important role in the acquisition of desiccation tolerance during the seed maturation of *Coffea canephora* (Stavrinides et al., 2020). Furthermore, the balance between hormones has been demonstrated to be able to induce desiccation tolerance in recalcitrant seeds, i.e., *Acer saccharinum* and *C. limon* seeds (Beardmone and Whittle, 2005; Marques et al., 2019). In the present study, desiccation leads to the differential expression of genes related to hormone biosynthesis and catabolism. The content of ABA and IAA were changed. Therefore, the desiccation sensitivity of *Q. variabilis* seeds might be a result of broken balance between ABA and IAA. Interestingly, it was found that there was a significantly negative correlation between IAA and seed germination. It might give a hint for inducing desiccation tolerance of *Q. variabilis* seeds in the future.

### Plant Hormone Signal Transduction

Plants can respond to stress by regulating the expression of genes in the ABA signaling pathway, which is critical for plant response to water deficit (Beardmone and Whittle, 2005). The ABA receptors PYR/PYLs, PP2Cs, SnRK2s, and ABF constitute the core network of ABA signal regulation (Zhu, 2016; Chen et al., 2019). In plants, the perception of ABA through PYR/PYL receptors is necessary for ABA signal transduction. The PYR can interact with ABA, inactivate PP2C, activate SnRK2, and self-phosphorylation, activate ABF, and then regulate the expression of related stress genes (Umezawa et al., 2009; Yoshida et al., 2014). During desiccation, the downregulation of PYL, PP2C, and SnRK may cause recalcitrant tea seeds sensitive to desiccation (Jin et al., 2018). In the study, desiccation induced downregulation of PYR, PP2C, and ABF, while SnRK2 were both up and downregulated. These results indicate that desiccation interferes with the ABA signal transduction pathway, which eventually results in high desiccation sensitivity.

The TIR1/AFB-AUX/IAA/TPL-ARFs pathway is a widely accepted IAA signaling pathway. IAA can promote the formation of synergistic receptor complexes between TIR1 and AUX/IAA proteins, thereby activating TFs of auxin response factor (ARF) family and regulating IAA response genes (Tan et al., 2007). In the present study, the expression of AUX/IAA genes were both up and downregulated, that of the ARF genes were downregulated and SAUR gene was up regulated, similar to the findings in *C. canephora* seeds (Stavrinides et al., 2020). It indicates that IAA signaling pathway might contribute to regulate seed desiccation sensitivity.

The transcription factors of *ERF1B, ABI5, TGA9, TGA10, UNE10, PIF3*, and *PIF5* were involved in “Plant hormone signal transduction”. Among them, ERF1B protein play an important role in tolerance to abiotic stress (Zhang et al., 2009). Furthermore, it has been reported that *ABI3* is essential for acquisition of seed desiccation tolerance (Khandelwal et al., 2010; González-Morales et al., 2016). The *ABI3* can interact with *ABI5* to regulate the expression of downstream genes and mediate ABA signal transduction (Delahaie et al., 2013). In the research, the *ERF1B* was up regulated and *ABI5* was downregulated, which may change the expression of downstream stress-related genes and result in seed desiccation sensitivity. These TFs need to be further explored, in order to provide reference for understanding the molecular regulatory mechanism of seed desiccation sensitivity.

### Protective Proteins Related to Desiccation

HSPs play irreplaceable roles in protecting plants from abiotic stress (Wang et al., 2004) and are particularly related to the acquisition of seed desiccation tolerance (Marques et al., 2019). There is a 22 kDa HSPs in recalcitrant *Acer saccharinum* seeds and its content increases significantly after desiccation, so as to reduce the damage (Kalemba and PukaControla, 2012). In this research, eight kinds of HSPs were identified with molecular weight of 15.7, 17.5, 18.1, 18.2, 18.5, 22.0 and 22.7 kDa, as well as HSP70, which were mainly up regulated during desiccation. This result is consistent with recalcitrant *T. chinensis* seeds (Wei et al., 2017). In addition, LEAs and the transcripts encoding LEAs were reported to accumulate in large amounts during acquisition desiccation tolerance of orthodox seeds (Giarola et al., 2017), which can prevent protein aggregation during desiccation by folding (Battaglia et al., 2008). While recalcitrant seeds have fewer LEAs or the absence of specific LEAs (Berjak and Pammerter, 2008; Delahaie et al., 2013). In this study, LEA-related genes were mainly downregulated during seed desiccation, which may inhibit the capability of *Q. variabilis* seeds to accumulate LEAs to resist the damage. Furthermore, ABI3 indirectly induced the desiccation tolerance of seeds by regulating the expression of HSPA9 to increase HSPs content (Verdier et al., 2013). The ABI5 and ABI3 also connect many LEAs related genes in *M. truncatula* seeds (Verdier et al., 2013). In the present study, ABI5 may regulate the expression of genes related to HSPs and LEAs cope with the adverse effects of desiccation. However, the specific regulatory relationship between them needs further experimental analysis.

### Glycerophospholipid Metabolism

Membrane phospholipids proportions are different between recalcitrant and orthodox seeds (Pukacka, 1999; Liu et al., 2006). Phospholipase D (PLD) can hydrolyze phospholipids into phospholipid acid (PA) and is closely related to desiccation sensitivity, i.e., the increase of PA content and decrease of seed survival under desiccation (Chen et al., 2018). Furthermore, PA also can be induced by ABA and as the second messenger to participate in plant responses to abiotic stress (Zhu, 2016). In this study, several ABA-related genes were differentially expressed. It was also found that PLD related genes were up regulated. Therefore, PLD may become more abundant and increase PA content during desiccation, resulting in reduction of the membranes fluidity and sensitivity to the desiccation of *Q. variabilis* seeds. In addition, the enhanced level of glutathione likely reduced the activity of PLD, suggesting it can improve *Acer saccharinum* seeds recalcitrance (Kalemba and Ratajczak, 2018). For *Arabidopsis* seeds, the poor germinability under oxidative conditions, apparently due to lower glutathione and cysteine contents (Cohen et al., 2014). In the research, it was found that “Cysteine and methionine metabolism” and “Glutathione metabolism” pathway was significantly enriched during desiccation. They may play roles in response to desiccation of *Q. variabilis* seeds. This is an issue that merits further investigation in the future.

**Figure 11:**
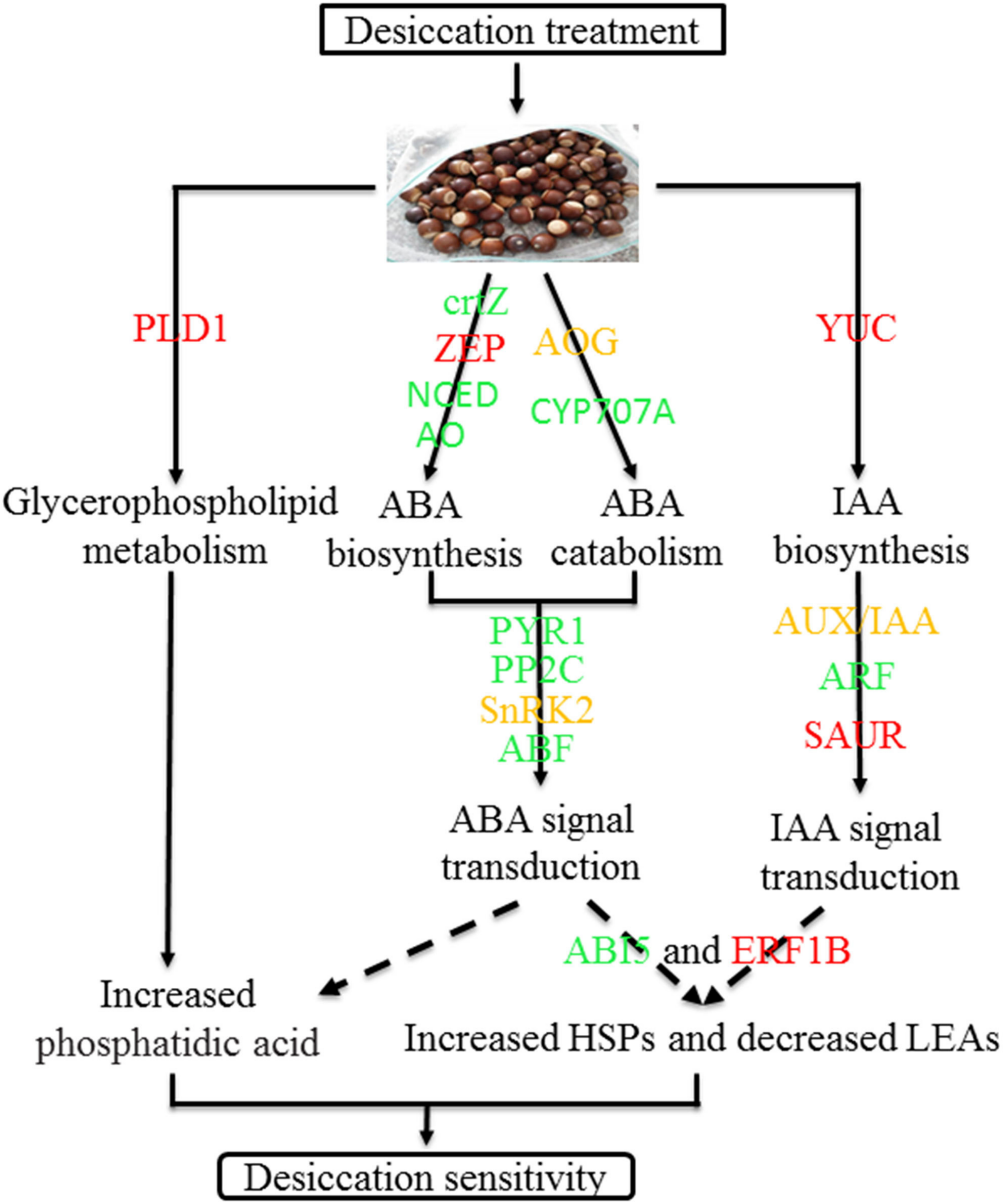
A model for mechanisms underlying desiccation sensitivity of *Q. variabilis* seeds. Solid lines indicate activation, and dashed lines indicate postulated regulation. Red indicates that the gene expression is up regulated, green indicates that the gene expression is downregulated, while orange indicates that there are both up regulated and downregulated genes in the gene family.

In conclusion, desiccation leads the change of ABA and IAA content and there was significantly negative correlation between IAA and seed germination. During the desiccation process, many genes related to hormone biosynthesis and signal transduction were differentially expressed, such as *GLOX, NCED1, PP2C77, PYR1, ARF18*, etc. Furthermore, several TFs and protective protein genes, including *ABI5, ERF1B, LEA D-29, HSP70*, etc., were also identified which may play important roles in response to desiccation of *Q. variabilis* seeds. Finally, a possible work model was proposed to show the molecular regulation mechanism of desiccation sensitivity in recalcitrant *Q. variabilis* seeds (Figure 11). The findings will contribute to the seed storage and long-term conservation of germplasm resources of recalcitrant seeds.

## Data Availability Statement

The datasets generated in this study are included in its Supplementary Materials. The raw RNA-Seq data for the four desiccation stages with three replicates are uploading in the NCBI Sequence Read Archive (SRA) repository via submission number PRJNA680427.

## Author Contributions

HY, LW, DL, and YL designed the experiments. DL, YL, and JQ performed the experiments. DL, GZ, and JR collected the materials. HX and XL coordinated the studies. DL wrote the manuscript. LZ and DL revised the manuscript. All authors have read and approved the final manuscript.

## Funding

This work was supported by the National Key Research and Development Program of Ministry of Science and Technology of China (No. 2017YFD0600602), the Fundamental Research Funds for the Central Non-profit Research Institution of CAF (CAFYBB2018ZB001), the open funding of State Key Laboratory of Tree Genetics and Breeding (TGB2019004), the Personnel Startup Project of the Scientific Research and Development Foundation of Zhejiang A&F University (2021FR041), and the Youth Top Talent Project of the Ten Thousand Talents Program of the State.

## Conflict of Interest

The authors declare that the research was conducted in the absence of any commercial or financial relationships that could be construed as a potential conflict of interest.

## Publisher's Note

All claims expressed in this article are solely those of the authors and do not necessarily represent those of their affiliated organizations, or those of the publisher, the editors and the reviewers. Any product that may be evaluated in this article, or claim that may be made by its manufacturer, is not guaranteed or endorsed by the publisher.

## Supplementary Material

Supplementary Figure S1 The correlated analysis between IAA and moisture contents of *Q. variabilis* seeds.

Supplementary Figure S2 The correlated analysis between IAA content and germination percentage of *Q. variabilis* seeds.

Supplementary Figure S3 The correlated analysis between ABA and moisture contents of *Q. variabilis* seeds.

Supplementary Figure S4 The correlated analysis between ABA content and germination percentage of *Q. variabilis* seeds.

Supplementary Figure S5 GO enrichment analysis of group specific DEGs.

Supplementary Figure S6 KEGG enrichment analysis of group specific DEGs.

Supplementary Table S1 Primer sequences for qRT-PCR.

Supplementary Table S2 Statistics of transcriptome sequencing data.

Supplementary Table S3 Differentially expressed genes in all three comparison groups of *Quercus variabilis* seeds during desiccation.

Supplementary Table S4 Group specific differentially expressed genes in *Quercus variabilis* seeds during desiccation.

Supplementary Table S5 Gene Ontology annotation of DEGs in *Quercus variabilis* seeds during desiccation.

Supplementary Table S6 Kyoto Encyclopaedia of Genes and Genomes annotation of DEGs in *Quercus variabilis* seeds during desiccation.
