# Supplementary material for: Comparative Transcriptome Analysis Revealed Candidate Genes Potentially Related to Desiccation Sensitivity of Recalcitrant Quercus variabilis Seeds
Source: Front Plant Sci. 2021 Sep 20;12:717563. doi: 10.3389/fpls.2021.717563 (PMC8488369; doi:10.3389/fpls.2021.717563)
Supplement: Supplementary Figure S1 — The correlated analysis between IAA and moisture contents of Q. variabilis seeds. [file Data_Sheet_1.ZIP › Supplementary Files/Supplementary Table S1 Primer sequences for qRT-PCR.docx]

SUPPLEMENTARY TABLE S1 Primer sequences for qRT-PCR

| Gene ID | Forward primer | Reverse primer |
| --- | --- | --- |
| ACT | GCTGGTCGTGATCTAACTG | CTTTGCAGTCTCCAACTCCT |
| LOC112024391; ABI5 | ACTCTTCCCCAAACCAACAAA | CTCCAACCACTGAACCCCTAC |
| LOC112005008; ARF18 | TTCCATCTTCTGAAATCACCACC | TGGACTTCAGCAGCACTACCTAA |
| LOC111992036; CYP707A | TCTTCTACACCACCTCACCACTT | GGGACAACTTTTATAGGCAAACC |
| LOC111996147; ERF1B | GCGGGGTTCCTCAGCTATAC | ATCCTCAAAGACCACCACATTC |
| LOC112025119; 70 kDa HSP | AAGTGGTTTTAGTAGGTGGGTCG | CGTAACATCTAGCAACAACAAGTCT |
| LOC111998831; LEA D-29 | GACACTGCGAAGGAAACGATG | ACTTTTCATAAGCCCAATCAGC |
| LOC112012911; probable NCED5 | ACTGTGCTTTACATTCTCCTTCG | TGTGCGTTCTTCTTGGGTTT |
| LOC112002222; PP2C77 | AGATGACTCCACCCATTTCCTT | AACTTGAGCCCATTGATTTCCT |
| LOC112010754; FMO GS-OX-like | GTTTTCCCGCCACAGTTAGC | TAGAGTTCTTCAACAGATGATGCC |
