# Supplementary material for: Comparative Transcriptome Analysis Revealed Candidate Genes Potentially Related to Desiccation Sensitivity of Recalcitrant Quercus variabilis Seeds
Source: Front Plant Sci. 2021 Sep 20;12:717563. doi: 10.3389/fpls.2021.717563 (PMC8488369; doi:10.3389/fpls.2021.717563)
Supplement: Supplementary Figure S1 — The correlated analysis between IAA and moisture contents of Q. variabilis seeds. [file Data_Sheet_1.ZIP › Supplementary Files/Supplementary Table S2 Statistics of transcriptome sequencing data.docx]

SUPPLEMENTARY TABLE S2 Statistics of transcriptome sequencing data

| Sample | Raw reads | Clean reads | Total mapped | Uniquely mapped | Mapped ratio (%) | Q30(%) | GC content (%) |
| --- | --- | --- | --- | --- | --- | --- | --- |
| CK-1 | 52921904 | 50513170 | 44216681 | 26315733 | 87.53 | 98.23 | 44.00 |
| CK-2 | 54685384 | 51549110 | 44983651 | 26670227 | 87.26 | 98.13 | 44.00 |
| CK-3 | 51137940 | 48480550 | 42824131 | 25507187 | 88.33 | 98.28 | 44.00 |
| T2-1 | 54435374 | 52483094 | 45894610 | 26922725 | 87.45 | 98.11 | 44.00 |
| T2-2 | 54696364 | 50785982 | 44047583 | 26277505 | 86.73 | 98.14 | 43.50 |
| T2-3 | 51382704 | 48438116 | 42083575 | 24718060 | 86.88 | 98.16 | 44.00 |
| T4-1 | 49107396 | 46640728 | 40614707 | 23904775 | 87.08 | 97.90 | 43.50 |
| T4-2 | 53360406 | 50887508 | 44494104 | 26134777 | 87.44 | 97.86 | 44.00 |
| T4-3 | 51038360 | 48254778 | 42142034 | 24529806 | 87.33 | 97.85 | 44.00 |
| T11-1 | 53581824 | 50834226 | 44477363 | 26358250 | 87.49 | 98.05 | 44.00 |
| T11-2 | 50996304 | 47851220 | 41841824 | 24641342 | 87.44 | 98.07 | 43.50 |
| T11-3 | 52101884 | 49448056 | 43360526 | 25904096 | 87.69 | 98.08 | 43.50 |
